# Resveratrol Alleviates NEFA-Induced Oxidative Damage in Bovine Mammary Epithelial Cells by Restoring Mitochondrial Function

**DOI:** 10.3390/ani15020118

**Published:** 2025-01-07

**Authors:** Longwei Sun, Junpeng Huang, Xiangyang Dou, Zhenyu Dong, Yuan Li, Shujing Tan, Ran Yu, Chengmin Li, Weiguo Zhao

**Affiliations:** 1 Jiangsu Key Laboratory of Sericultural and Animal Biotechnology, School of Biotechnology, Jiangsu University of Science and Technology, Zhenjiang 212100, China; sunlongwei163@163.com (L.S.); jaime27@163.com (J.H.); 18115194692@163.com (X.D.); 15061006910@163.com (Z.D.); liyuan981225@163.com (Y.L.); tanshujing2023@126.com (S.T.); 18912250570@163.com (R.Y.); 2 Key Laboratory of Silkworm and Mulberry Genetic Improvement, Ministry of Agriculture and Rural Affairs, The Sericultural Research Institute, Chinese Academy of Agricultural Sciences, Zhenjiang 212100, China

**Keywords:** resveratrol, NEFA, oxidative damage, mitochondria, bovine mammary epithelial cells

## Abstract

In periparturient dairy cows, high non-esterified fatty acids (NEFAs) caused by a severe negative energy balance induce oxidative stress and metabolic dysfunction, which pose a severe challenge to the dairy industry. Resveratrol (RES) is a polyphenolic compound with antioxidant, anti-inflammatory and multiple other physiological effects. However, its effect on oxidative damage triggered by NEFAs in bovine mammary epithelial cells is rarely reported. This study aimed to investigate the antioxidant effects and underlying molecular mechanisms of RES in NEFA-challenged BMECs. The results showed that RES ameliorated NEFA-induced oxidative damage by upregulating antioxidant enzyme expression and reducing malondialdehyde (MDA) and reactive oxygen species (ROS). Furthermore, exogenous NEFAs resulted in a decrease in mitochondrial membrane potential (MMP), cellular adenosine triphosphate (ATP) production, energy metabolism (NAD^+^/NADH ratio), abnormal mitochondrial structure and an increase in apoptosis levels. RES treatment restored mitochondrial function in NEFA-stressed BMECs, as evidenced by the increase in MMP, ATP generation and NAD^+^/NADH ratio accompanying the decline in mitochondrial structural abnormalities and cell apoptosis. In addition, in vivo studies in a mouse model of oxidative damage induced by high-fat diet (HFD) demonstrated that RES alleviated oxidative damage (decreased MDA content) and mitochondrial dysfunction (decreased expression of Drp1 and Fis1 and increased levels of Mfn2, Cyt C mRNA and ATP production) in mammary gland tissue. Overall, these findings suggested that RES could alleviate NEFA-induced oxidative damage in BMECs by modulating mitochondrial function, thereby contributing to the prevention and treatment of oxidative damage in perinatal dairy cows with a negative energy balance.

## Simple Summary

Resveratrol (RES), a polyphenolic compound, is known for its diverse bioactivities such as its antioxidant and antimicrobial properties. This study explored the antioxidant effects of RES on NEFA-induced BMECs. It focused on mitochondrial function using both in vitro and in vivo approaches. The results showed that RES effectively reduced oxidative stress, cell death and mitochondrial damage caused by elevated NEFA levels in bovine mammary cells, and enhanced antioxidant potential and mitochondrial function in mammary gland tissue. These findings suggest that RES could mitigate oxidative damage by modulating mitochondrial function.

## 1. Introduction

The perinatal period, from 3 weeks before to 3 weeks after parturition, is the most critical period in the productive life of dairy cows [1,2]. During this period, a state of negative energy balance (NEB) is the most notable physiological characteristic, and this delicate balance between energy availability and consumption puts the cows into states of physiological changes and immune suppression [3,4,5,6]. Furthermore, severe negative energy balance can lead to the occurrence of oxidative stress, which can reduce the cow’s immunity throughout the delivery process, thereby increasing the risk of infectious and metabolic diseases [7]. In particular, mastitis, the most frequent, costly and complex disease of dairy cows, poses a great threat to the development of the dairy industry and brings huge economic losses.

It has been ascertained that NEB could trigger lipid mobilization and a subsequent high circulating concentration of free fatty acids (NEFAs), which is a crucial precipitating factor for metabolic and infectious diseases during the transition period [8,9]. Clinical and experimental data from previous studies indicate that high levels of NEFAs are closely related to an increased incidence of certain diseases, such as mastitis, endometritis and ketosis in dairy cows, which adversely affect the health, longevity, productive and reproductive performances of dairy cows [10,11,12,13]. Furthermore, elevated levels of NEFAs during this period significantly increase the accumulation of ROS, thereby triggering oxidative stress and causing cellular dysfunction, ultimately affecting the health of the animal [14]. In addition, mitochondria, serving as the energy centers of cells, not only provide the energy required for cellular life but also are the primary sources of cellular ROS production. Previous research has shown that high concentrations of NEFAs possess lipotoxicity, which induces ROS production via oxidative phosphorylation, eventually causing mitochondrial dysfunction, inducing the release of pro-apoptotic factors and triggering apoptosis [15,16]. Thus, exploring strategies to protect transition dairy cows from the oxidative damage caused by NEFAs is particularly important.

Resveratrol, a natural polyphenolic compound, was originally thought to be a phytoalexin and primarily found in mulberry leaves, peanuts, and some other plants. It has the properties of low aqueous solubility, poor bioavailability and rapid absorption [17]. Previous research has demonstrated that resveratrol exhibits a diverse array of biological activities, such as antioxidation, anti-inflammation, microbiome modulation and anti-aging [18,19,20]. Multiple studies, both in vivo and in vitro, have shown that the anti-inflammatory effects of resveratrol probably function via suppressing the anti-inflammatory factors production [21]. Recently, a study reported that RES can enhance the activity of antioxidant enzymes, thereby maintaining the homeostasis of ROS in hydrogen peroxide-induced porcine intestinal epithelial cells [22]. On the other hand, RES can improve mitochondrial biogenesis and redox status by regulating the activity of SOD and GSH-Px in suckling piglets [23]. Additionally, RES could enhance the antioxidant capacity and production performance of laying hens [24]. Therefore, we hypothesize that resveratrol could mitigate oxidative damage and enhance mitochondrial function in NEFA-stimulated BMECs. The present study aimed to investigate the antioxidant effects and underlying molecular mechanisms of RES in NEFA-induced BMECs, with a focus on mitochondrial function both in vitro and in vivo.

## 2. Materials and Methods

### 2.1. Cell Culture and Treatments

The bovine mammary epithelial cell line (BMECs, MAC-T) was cultured in DMEM/F12 medium (Gibco, Grand Island, NY, USA), supplemented with 10% fetal bovine serum (Zeta Life, Menlo Park, CA, USA) and 1% penicillin/streptomycin (Invitrogen, Carlsbad, CA, USA), and maintained at 37 °C in 5% CO_2_. The medium was refreshed regularly. BMECs were treated with NEFAs (0.9 mM, 4 h) and various concentrations of RES (15–200 µM, 24 h). The NEFA and RES dose were selected based on our previous studies and a preliminary experiment [25,26]. RES was prepared in DMSO and diluted to working concentrations as described. For control groups, equivalent volumes of DMSO were added. The NEFA stock solution (50 mM) comprised 7.2 mM stearic acid, 2.45 mM linoleic acid, 2.65 mM palmitoleic acid, 21.75 mM oleic acid and 15.95 mM palmitic acid.

### 2.2. Cell Viability Assay

BMECs were seeded into 96-well plates and treated with different concentrations of resveratrol for 24 h; after pretreatment of BMECs with resveratrol for 20 h, cells were then treated with 0.9 mM NEFAs for 4 h. Following the treatment, 10 μL of CCK-8 reagent was added to each well and incubated for 4 h. Next, according to the manufacturer’s instructions (CCK-8, Beyotime, Shanghai, China), we measured the absorbance at a wavelength of 450 nm using a microplate reader.

### 2.3. Intracellular Reactive Oxygen Species Measurement

After treatment, BMECs were washed three times with PBS and incubated with 10 mM DCFH-DA for 20 min using a ROS detection kit (Beyotime, Shanghai, China), with gentle mixing every 3–5 min. The cells were then resuspended in PBS and immediately analyzed under a fluorescence microscope (Olympus, Tokyo, Japan). Intracellular ROS levels were quantified as relative fluorescence, normalized to the control group.

### 2.4. Measurement of Mitochondrial Membrane Potential

The fluorescent probe JC-1 (Beyotime, Shanghai, China) was used as an assay for mitochondrial membrane potential changes. BMECs were incubated with JC-1 staining buffer for 20 min at 37 °C, washed twice with JC-1 staining buffer (1×), and then observed by fluorescence microscopy and analyzed by ImageJ software (Version 1.53t, National Institutes of Health, Bethesda, MD, USA).

### 2.5. Transmission Electron Microscopy

Cells were co-cultured with 100 μM RES for 24 h, with or without 0.9 mM NEFAs for 4 h. After treatment, cells were collected and fixed in 2.5% glutaraldehyde for 4 h, followed by 1% osmium tetroxide (OsO_4_) for 1 h. The cells were dehydrated through a graded ethanol series and infiltrated with propylene oxide resin. Ultrathin cell sections were stained with uranyl acetate and lead citrate, and the ultrastructure was observed using transmission electron microscopy (TEM, Hitachi, Tokyo, Japan).

### 2.6. Flow Cytometer Detection of Apoptosis

In this study, apoptosis was assessed using the Annexin V-FITC/PI Detection Kit (Beyotime, Beijing, China), followed by flow cytometry (FCM) using a FACSCalibur (BD Biosciences, Bedford, MA, USA). In brief, BMECs were treated with specified concentrations of NEFAs and RES. After treatment, the cells were collected, washed three times with PBS, and incubated for 25 min at room temperature in the dark, according to the manufacturer’s instructions. Cellular fluorescence was then measured using a FACS Calibur flow cytometer (BD Biosciences, Bedford, MA, USA), and data were analyzed with FlowJo software (Version 9, Becton, Dickinson and Company, Franklin Lakes, NJ, USA).

### 2.7. Animals and Experimental Design

A total of 40 BALB/c mice (aged 6–8 weeks, 20–25 g) were purchased from the Laboratory Animal Center of Jiangsu University. All mice were given free access to food and water and housed under standard conditions (12 h light/dark cycle, relative humidity of 55 ± 5%, and a temperature of 22–23 °C). After a one-week acclimation period, mice were randomly divided into five groups (*n* = 8 per group): (1) The control (CON) group were fed with a basal diet. (2) The high-fat diet (HFD) group received a diet containing 45% energy from fat. (3) The RES30 + HFD group were fed with HFD and orally administered 30 mg/kg/day of RES (purity 98%; Chengdu Herbpurify Co., Ltd., Chongqin, China; diluted in 0.5% CMC-Na). (4) The RES60 + HFD group were fed with HFD and orally administered 60 mg/kg/day of RES. (5) The RES120 + HFD group were fed with HFD and orally administered 120 mg/kg/day of RES. The doses of resveratrol were selected based on previous studies [27,28]. Mice growth was monitored daily, and pregnant mice were housed separately. No mortality was observed in the HFD or RES treatment groups during the experimental period.

### 2.8. Sample Collection

After 10 weeks of feeding, on days 9 to 14 of lactation, mice were fasted for 12 h and euthanized using cervical dislocation. Blood was collected via orbital enucleation, centrifuged at 3500 rpm for 10 min at 4 °C and then stored at −80 °C. The fourth pair of mammary glands were immediately excised, washed in neutral-buffered saline, and trimmed of excess tissue. A portion of the mammary glands were fixed in 4% paraformaldehyde for histopathological analysis, while the remaining tissues were frozen in liquid nitrogen and then stored at −80 °C until analysis.

### 2.9. Histological Observation

As previously reported [29], mammary glands were fixed in 4% paraformaldehyde and embedded in paraffin. After paraffin embedding, the mammary glands were sectioned into 5 μm thick slices and stained with hematoxylin and eosin (H&E) to evaluate structural changes. Histological observations were conducted using a light microscope to assess structural alterations (Olympus, Tokyo, Japan). and analyze inflammatory cell infiltration.

### 2.10. Immunohistochemistry (IHC) Analysis

Tissue samples were fixed in 4% paraformaldehyde for 24–48 h, followed by paraffin embedding and sectioning at a thickness of 4 μm. After deparaffinization, the mammary tissue sections underwent antigen retrieval and were incubated in PBS containing 5% bovine serum albumin (BSA) for 1 h. The sections were then incubated overnight at 4 °C with primary antibody DRP1 (1:300, Servicebio, Wuhan, China). After washing, the sections were incubated at room temperature for 1 h with a fluorescent secondary antibody (1:200, Servicebio, Wuhan, China). Finally, DAPI staining solution was applied, and the sections were incubated for 10 min in the dark at room temperature. The images were captured using a fluorescence microscope (Olympus, Tokyo, Japan).

### 2.11. TUNEL Detection

Mouse mammary glands were fixed in 4% paraformaldehyde and embedded in paraffin. The paraffin blocks were then sectioned into slices 2–3 microns thick. TUNEL staining was performed following the manufacturer’s instructions using a TUNEL apoptosis detection kit (Servicebio, Wuhan, China). TUNEL-positive cells were observed under an optical microscope (Olympus, Tokyo, Japan).

### 2.12. Determination of Oxidative Stress Indicators

According to the manufacturer’s instructions, commercial biochemical analysis kits (Nanjing Jiancheng Bioengineering Institute, Nanjing, China) were used to measure the activities of total superoxide dismutase (T-SOD) and glutathione peroxidase (GSH-Px), as well as the content of malondialdehyde (MDA) in the cell supernatant and plasma. Absorbance was measured using a microplate reader at wavelengths of 560 nm (SOD), 420 nm (GSH-Px), 405 nm (CAT) and 532 nm (MDA).

### 2.13. Determination of ATP Content

The ATP content in mammary tissues and cells was measured using an ATP Assay Kit (Beyotime, Shanghai, China) following the manufacturer’s instructions. Tissues and cells were lysed with lysis buffer provided by the manufacturer, and the supernatants were collected by centrifugation at 12,000× *g* for 10 min at 4 °C. The ATP levels were normalized to protein content, and the results were expressed as relative ATP content compared to the control group.

### 2.14. NAD^+^/NADH Ratio Assay

According to the manufacturer’s instructions (Beyotime, Shanghai, China), the levels of NAD^+^ and NADH in the BMECs and mammary tissues were assessed using an NAD^+^/NADH assay kit. We mixed 20 μL of supernatant with the ethanol dehydrogenase working solution, then incubated it in the dark at room temperature for 15 min. Finally, absorbance was determined by a microplate reader (BioTek, Shoreline, WA, USA) at wavelengths of 450 nm. By subtracting NADH from the total NAD^+^/NADH, the amount of NAD^+^ was derived.

### 2.15. Quantitative Real-Time PCR Analysis

Total RNA was extracted from mammary tissues using Trizol reagent (15596026, Invitrogen, Carlsbad, CA, USA) following the manufacturer’s protocol. Complementary DNA (cDNA) was synthesized using the PrimeScript RT Reagent Kit (TaKaRa, Otsu, Japan). Quantitative real-time PCR (qPCR) was performed using SYBR Premix Ex Taq™ (TaKaRa, Otsu, Japan) on an Applied Biosystems 7500 HT Sequence Detection System. The PCR program consisted of an initial denaturation step at 95 °C for 30 s, followed by 40 cycles of 95 °C for 5 s and 60 °C for 34 s, with fluorescence signal collection at 60 °C. The following primer sequences were used: Cyt C-Forward: 5′-AGCAAGCTTCAGCGAAGATG-3′, Cyt C-Reverse: 5′-GGAAGGCAGCAAAGATGACA-3′. Fis1-Forward: 5′-AGATGGAGCAAGGAGGAGTG-3′. Fis1-Reverse: 5′-GGTCCACAGTGGTGTCAGAT-3′. Mfn2-Forward: 5′-TTGAAATGCGGGAAGAG-3′. Mfn2-Reverse: 5′-CCTTTCCACTTCCTCTG-3′. Drp1-Forward: 5′-CCACTCGGACTGCCTTCT-3′. Drp1-Reverse: 5′-CTGCTCCCCACATCAACA-3′. GAPDH-Forward: 5′-CCACCCATGGCAAATTCCATGGCA-3′. GAPDH-Reverse: 5′-TCTAGACGGCAGGTCAGGTCCACC-3′. All primer pairs in this study were designed based on gene sequences from GenBank using Primer Express software (Version 3.0, Applied Biosystems Inc., Foster City, CA, USA). The primers were then synthesized by Shangya Biotechnology Co., Ltd. (Jiaxing, China). The experiment was conducted in triplicate, and data were analyzed using the 2^−ΔΔCT^ method.

### 2.16. Western Blotting Analysis

Mammary tissues and cells were homogenized using RIPA lysis buffer. After centrifugation at 12,000 rpm for 15 min at 4 °C to determine the protein concentration of each sample, the supernatant was assayed for protein concentration using a BCA protein test kit (Beyotime, Shanghai, China). Proteins were separated via sodium dodecyl sulfate–polyacrylamide gel electrophoresis (SDS-PAGE) and transferred onto a polyvinylidene fluoride (PVDF) membrane. The membranes were blocked with 5% skim milk in Tris-buffered saline with Tween (TBST) at room temperature for 2 h, followed by overnight incubation at 4 °C with the following primary antibodies: rabbit anti-Bcl2 (1:4000, Proteintech, Rosemont, IL, USA, USA12789-1-AP), rabbit anti-SOD2 (1:1000, Proteintech, Rosemont, IL, USA, USA24127-1-AP), rabbit anti-Bax (1:5000, Proteintech, Rosemont, IL, USA, USA50599-2-lg), rabbit anti-caspase-3 (1:1000, ABclonal, Woburn, MA, USA, USAA11021), and rabbit anti-Tubulin (1:1000, Bioworld, Bloomington, MN, USA). The blots were incubated with HRP-anti-mouse antibodies or conjugated anti-rabbit (1:5000, Proteintech, Rosemont, IL, USA), and the signals were detected by enhanced chemiluminescence (ECL) Western blot detection reagents (Pierce, Rockford, IL, USA). Finally, signals were quantified by ImageJ software (National Institutes of Health, Bethesda, MD, USA).

### 2.17. Statistical Analysis

Statistical analyses were conducted using GraphPad Prism 9.5.0 software (GraphPad Software, San Diego, CA, USA). Data are presented as mean ± standard error of mean (SEM), and all experiments were performed in triplicate with three independent repeats. Both in vitro and in vivo data were analyzed using a consistent statistical approach. Data were examined for normality and homogeneity of variance by the Shapiro–Wilk and Levene tests. Differences between groups were analyzed using one-way analysis of variance (ANOVA), followed by Tukey’s test for multiple comparisons. Western blot band intensity and fluorescence measurements were quantified with ImageJ. A *p*-value of less than 0.05 was considered statistically significant.

## 3. Results

### 3.1. Effects of Resveratrol on Cell Viability in BMECs

**Figure 1:**
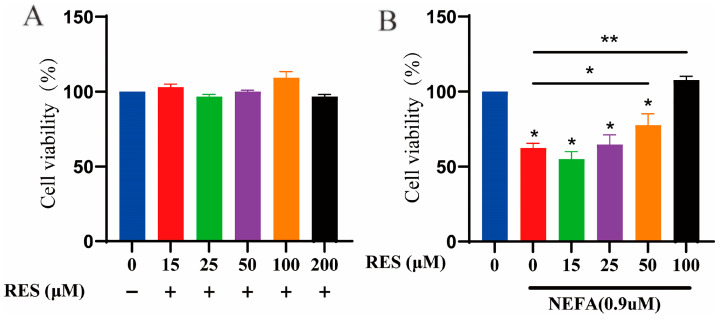
The effect of resveratrol on BMEC viability. (**A**) The viability of BMECs treated with different concentrations of resveratrol (15, 25, 50, 100 and 200 μM) for 24 h; (**B**) The effect of resveratrol on the viability of NEFA-treated BMECs. Data are presented as the means ± SEM of three independent experiments; * *p* < 0.05, ** *p* < 0.01.

The effects of different concentrations of RES (15, 25, 50, 100, 200 µM) and NEFAs (0.9 mM, 4 h) on the viability of BMECs were measured using the CCK-8 assay. The results showed that NEFAs significantly inhibited the growth of BMECs compared to the control group. After treating BMECs with different concentrations of RES for 24 h, it was found that RES treatment significantly enhanced the viability of BMECs, with the most pronounced effect at a concentration of 100 µM (Figure 1B).

### 3.2. Resveratrol Attenuated Oxidative Stress Caused by Elevated NEFA Levels in BMECs

**Figure 2:**
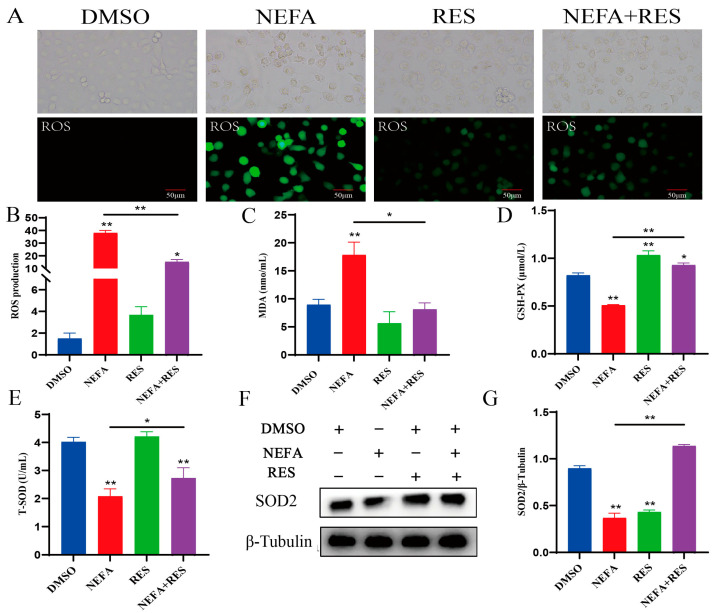
The effect of resveratrol treatment on oxidative stress in NEFA-stimulated BMECs. (**A**,**B**) The effect of resveratrol treatment on the levels of ROS in NEFA-stimulated BMECs, scale bar 50 μm; (**C**–**E**) the effect of resveratrol on the levels of MDA, GSH and SOD in BMECs treated with NEFA; (**F**,**G**) the effect of resveratrol on the protein expression of SOD2 in bovine mammary epithelial cells treated with NEFA. Data are presented as the means ± SEM of three independent experiments; * *p* < 0.05, ** *p* < 0.01.

The activities of ROS, SOD, MDA and GSH, as well as the protein expression levels of SOD2 were measured to evaluate the antioxidative performance of RES on NEFA-stimulated BMECs. As shown in Figure 2A,B, compared with DMSO group, ROS production significantly increased in the NEFA treatment group. However, RES significantly inhibited the accumulation of ROS triggered by NEFAs. Moreover, treatment with RES improved the antioxidant capacity in NEFA-challenged BMECs, as evidenced by the higher GSH and SOD levels and lower MDA content. These data suggest that RES exhibited antioxidant properties and could reduce NEFA-induced oxidative stress in BMECs.

### 3.3. Resveratrol Relieved Mitochondrial Dysfunction Induced by NEFA in BMECs

**Figure 3:**
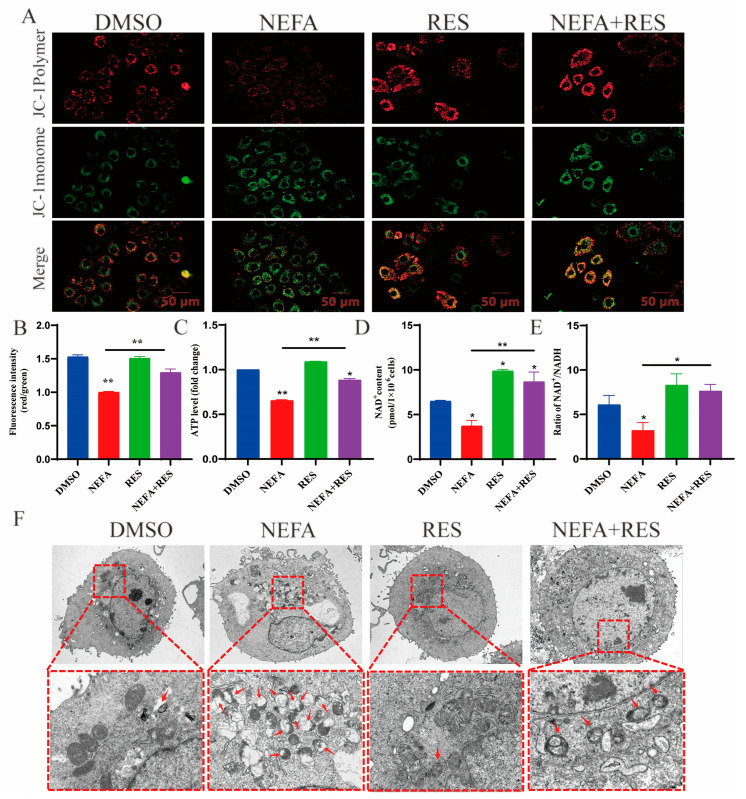
Effect of resveratrol on mitochondrial function in NEFA-induced BMECs. (**A**,**B**) Effect of resveratrol on MMP levels in NEFA-treated bovine mammary epithelial cells, with green and red fluorescence representing monomeric and aggregated forms of JC-1, respectively. Scale bar 50 μm. (**C**) Effect of resveratrol on ATP levels in NEFA-induced BMECs. (**D**,**E**) Effect of resveratrol on NAD^+^/NADH ratio in NEFA-induced bovine mammary epithelial cells. (**F**) Effect of resveratrol on ultrastructure of BMECs treated with NEFA, red arrowheads represent mitochondrial structural abnormalities. Data are presented as means ± SEM of three independent experiments; * *p* < 0.05, ** *p* < 0.01.

As shown in Figure 3A,B, it was evident that the MMP in the NEFA group was significantly reduced. Conversely, treatment with RES restored mitochondrial function in NEFA-stressed BMECs, as demonstrated by the improved MMP, increased ATP production and higher NAD^+^/NADH ratio (Figure 3A–E). Furthermore, TEM analysis revealed that RES mitigated NEFA-induced mitochondrial structural abnormalities such as swelling, cristae loss and vacuolization (Figure 3F). These results indicate that RES promoted mitochondrial function in NEFA-treated BMECs.

### 3.4. Resveratrol Attenuated Apoptosis Caused by Elevated NEFA Levels in BMECs

**Figure 4:**
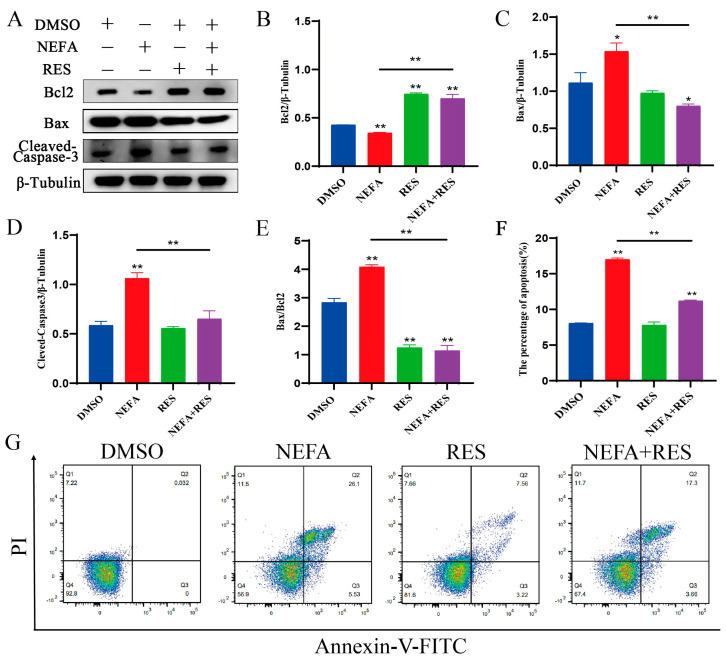
Effect of resveratrol on apoptosis of BMECs treated with NEFA. (**A**–**E**) Representative Western blots and quantitative assessment of apoptotic markers in BMECs treated with resveratrol and NEFAs; (**F**,**G**) effect of resveratrol on apoptosis rate of NEFA-treated BMECs. Data are presented as means ± SEM of three independent experiments; * *p* < 0.05, ** *p* < 0.01.

To further investigate the effects of resveratrol on oxidative damage triggered by NEFAs in BMECs, the expression of apoptosis-related proteins was measured. As shown in Figure 4A–E, Western blot results indicated that the Bcl-2 levels in the NEFA group were markedly decreased, while the levels of Bax and cleaved-caspase-3 were remarkably increased. Conversely, RES upregulated Bcl-2 expression and downregulated the levels of cleaved-caspase-3, Bax and the Bax/Bcl-2 ratio. Additionally, apoptosis was also detected by the Annexin V-FITC/PI double staining method; results showed that, compared with the NEFA group, RES treatment considerably reduced the apoptosis rate induced by NEFAs (Figure 4F,G). Collectively, these data demonstrate that RES could alleviate apoptosis triggered by NEFAs in BMECs.

### 3.5. Resveratrol Treatment Alleviates HFD-Induced Oxidative Damage in Mammary Glands of Mice

**Figure 5:**
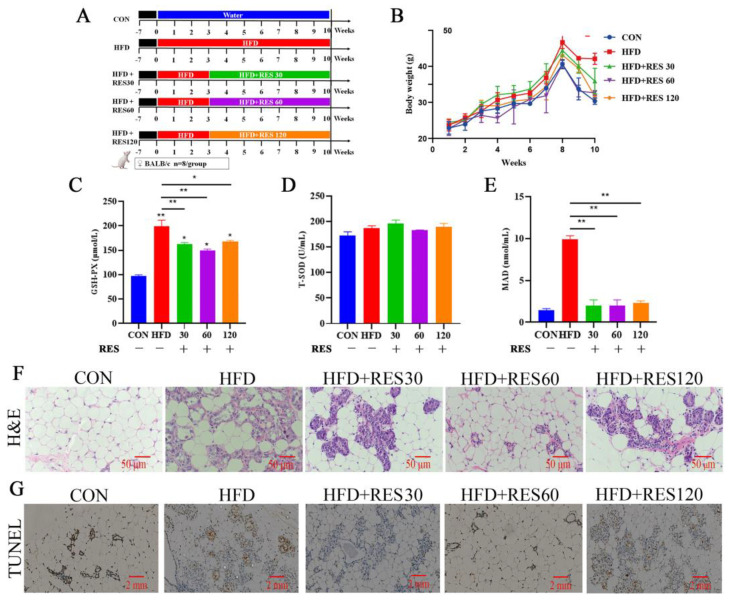
Effect of resveratrol on oxidative damage in mouse mammary glands when fed with HFD. (**A**) Schematic study design for the animal experiment; (**B**) body weight changes throughout this experiment; (**C**–**E**) effect of resveratrol on levels of GSH, SOD and MDA; (**F**) H&E staining of mammary glands; scale bar, 50 µm; (**G**) effect of resveratrol on apoptosis levels in mammary tissues analyzed by TUNEL staining; scale bar, 50 µm. Data are presented as means ± SEM of three independent experiments; * *p* < 0.05, ** *p* < 0.01.

The antioxidant effects of resveratrol were elucidated by investigating its impact on mammary tissue damage in HFD-fed mice. As shown in Figure 5B, the increase in body weight of the HFD-fed mice was reduced after 5 weeks of RES treatment. Furthermore, RES treatment showed a significant increase in GSH activity and decrease in MDA content when compared to the HFD group (Figure 5C,E). The H&E staining results showed that the high-fat diet significantly disrupted mammary tissue structure, with inflammatory cell infiltration and a disorganized alveolar structure, while RES treatment notably reduced the number of inflammatory cells, especially in the medium-dose group, and the alveolar structure was more intact (Figure 5F). Similarly, compared to the HFD group, the RES group showed a reduced level of cell apoptosis in mammary tissue (Figure 5G). Taken together, these results indicate that RES could alleviate HFD-induced oxidative damage in the mammary glands of mice.

### 3.6. Resveratrol Treatment Alleviates HFD-Induced Mitochondrial Dysfunction in Mammary Glands of Mice

**Figure 6:**
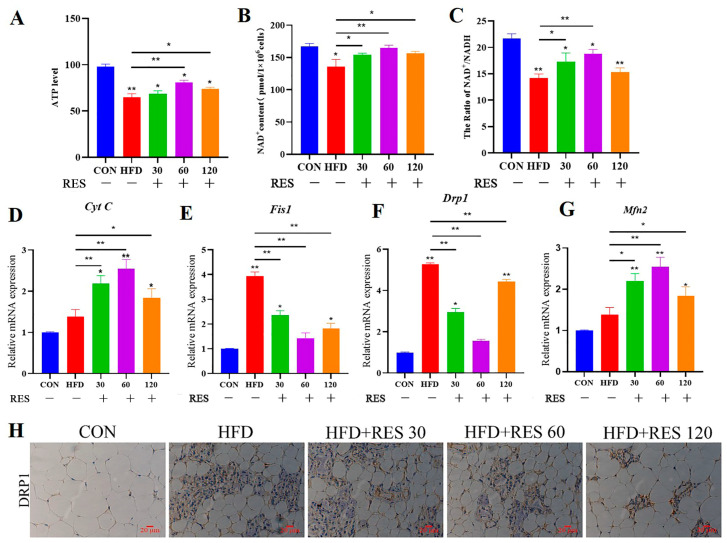
The effect of resveratrol on mitochondrial function in the mammary tissues induced with HFD. (**A**) The effect of resveratrol on the ATP levels in HFD-induced mammary tissues; (**B**,**C**) the effect of resveratrol on the NAD^+^/NADH ratio in HFD-induced mammary tissues; (**D**) the effect of resveratrol on DRP1 protein expression in HFD-induced mammary tissues; (**E**–**H**) the effect of resveratrol on the mRNA expression of Cytc, Fis1, DRP1 and MFN2 in HFD-induced mammary tissues. Data are presented as the means ± SEM of three independent experiments; * *p* < 0.05, ** *p* < 0.01.

Mitochondrial dysfunction leads to ROS accumulation, ATP consumption and subsequent energy depletion, disrupting intracellular homeostasis [30,31]. Thus, the role of RES on mitochondrial function in mammary tissues was further examined. The results showed that compared to the HFD group, RES treatment significantly increased the ATP levels and NAD^+^/NADH ratio (Figure 6A–C). Moreover, RES markedly increased the Mfn2 and Cyt C mRNA expression and reduced the mRNA expression levels of Fis1, and DRP1 in mouse mammary tissues (Figure 6D–G). Additionally, immunohistochemical staining revealed that the expression levels of DRP1 in the mammary tissue of HFD-treated mice were much higher than that of the control group. However, the RES treatment group showed significantly reduced DRP1 expression levels as compared to the HFD group (Figure 6H). These findings suggest that RES alleviated mitochondrial dysfunction in the mammary tissue of HFD mice.

## 4. Discussion

During the transition period, the mammary glands of high-lactation dairy cows undergo intense metabolic activity, rendering BMECs highly vulnerable to oxidative stress [32]. This oxidative stress causes significant damage to cellular structures and disrupts membrane integrity and functionality, which is a primary factor leading to reduced milk production and mammary gland injury [33]. Therefore, compounds with antioxidant properties may be beneficial in mitigating mammary oxidative stress. RES has been shown to possess antioxidant activity by enhancing antioxidant enzyme activity and curbing ROS production [22]. It can also protect MAC-T cells from oxidative stress and mitochondria-mediated apoptosis by scavenging ROS and activating the Nrf2-ARE self-defense mechanism [34]. Notably, resveratrol’s capacity to restore mitochondrial integrity is tightly coupled with its antioxidant role, as improved mitochondrial function can prevent excess ROS generation and maintain higher ATP levels [35]. Based on these bioactivities of RES, we conducted this study to explore the potential protective effect and mechanism of RES against NEFA-stimulated oxidative damage in BMECs.

The increased circulating levels of NEFAs caused by NEB can cause lipotoxicity and oxidative stress in dairy cows, thereby leading to a series of physiological changes, significantly reducing milk production and overall health [36,37]. Previous studies, along with our recent findings, have indicated that NEFAs can induce excessive ROS production and aggravate oxidative stress in bovine mammary epithelial cells [26,38,39]. Therefore, maintaining intracellular redox balance and reducing oxidative stress-induced diseases during the perinatal period in dairy cows is crucial. Recent studies have shown that RES possesses antioxidant activity and can reduce oxidative stress damage [20,40]. It was indicated that RES can reduce ROS generation in calf hepatocytes and bovine mammary epithelial cells [34,40]. Moreover, RES has been shown to inhibit aflatoxin B1-induced oxidative stress and apoptosis in MAC-T cells [41]. Consistent with these findings, our results demonstrated that RES not only significantly inhibited NEFA-challenged ROS production in BMECs but also increased antioxidant enzymes activities, indicating that RES has a protective effect against NEFA-stimulated oxidative stress in BMECs.

Mitochondria are essential organelles responsible for cellular oxidation and ATP production, serving as the main source of 90% of the cell’s ATP. Studies have shown that excessive ROS could damage mitochondria and affect their structure and proteins, ultimately leading to mitochondrial dysfunction [42]. During the perinatal period, mitochondrial dysfunction occurs in the mammary gland of NEB cows, and previous studies have demonstrated that elevated levels of NEFAs can cause mitochondrial dysfunction in BMECs [37,43]. RES has been reported to improve mitochondrial function in vitro and in vivo [44,45,46]. Consistent with these results, our findings indicated that RES relieved NEFA-induced mitochondrial damage through multiple mechanisms, including the restoration of mitochondrial membrane potential, enhanced ATP production and the rebalancing of NAD⁺/NADH levels, thereby strengthening the antioxidant defense within BMECs. Furthermore, RES significantly reduced the number of abnormal mitochondria, with the mitochondrial structure appearing clearer. These results provide new insights into the protective mechanisms of RES on NEFA-challenged BMECs by restoring mitochondrial function.

When mitochondria are damaged, ATP production is suppressed, leading to an insufficient cellular energy supply and decreased mitochondrial membrane potential (MMP) [47]. Cytc is then released from the intermembrane space into the cytosol, triggering the caspase cascade, activating cleaved-caspase-3, and ultimately initiating apoptosis. Furthermore, Bcl2 and Bax, members of the Bcl2 protein family, are key regulators in the early stages of the apoptotic pathway, penetrating the outer mitochondrial membrane to promote Cytc release [48,49]. In this study, RES inhibited the expression of the pro-apoptotic proteins Bax and cleaved-caspase-3 in NEFA-challenged BMECs, which is partly consistent with the previous studies on the role of RES in the aflatoxin B1-induced apoptosis of BMECs [41]. These results suggest that RES mitigated NEFA-induced apoptosis in BMECs by mediating the mitochondrial pathway.

Considering that the transition cows under NEB statu is a complicated process—the body fat is broadly broken down leading to the abnormal accumulation of NEFA, represented by palmitic acid (PA)—no appropriate in vivo model has yet been designed to replicate such a state. Moreover, because of the animal welfare policy and a limited experimental budget, and due to similarities between mice and cows, as an alternative, a mouse model of HFD was therefore used for in vivo studies to further confirm the protective effects of resveratrol against oxidative damage. Previous studies have indicated that RES significantly alleviated the oxidative stress in HFD-fed mice, rats and rabbits [50,51,52]. In this study, RES reduced the body weight of HFD-fed mice and preserved the structural integrity of alveoli, which was evidenced by the increased GSH and SOD levels and reduced MDA levels. In addition, recent research has reported that RES ameliorated HFD-induced mitochondrial dysfunctions in rats [53,54]. Consistent with the above studies, we found that RES positively affected the expression of mRNAs related to mitochondrial function (Drp1, Fis1, Cytc and Mfn2) in the mammary gland, and significantly increased ATP production and the NAD^+^/NADH ratio. Furthermore, RES decreased the number of TUNEL-positive cells, which was partly consistent with the research results obtained previously [55,56].

## 5. Conclusions

In conclusion, we demonstrated that the exogenous stimulation of NEFAs induces oxidative stress and mitochondrial dysfunction in BMECs, thereby leading to cell apoptosis. RES treatment reduced oxidative stress and further preserved mitochondrial function in both NEFA-treated BMECs and HFD-fed mice, which together alleviated cell apoptosis. These results suggest that RES could act as a potential therapeutic target for maintaining oxidative homeostasis in transition dairy cows experiencing a state of negative energy balance.

## Data Availability

All data presented in this study are available on request from the corresponding authors.
